# The lived experiences of adults with attention-deficit/hyperactivity disorder: A rapid review of qualitative evidence

**DOI:** 10.3389/fpsyt.2022.949321

**Published:** 2022-08-11

**Authors:** Callie M. Ginapp, Grace Macdonald-Gagnon, Gustavo A. Angarita, Krysten W. Bold, Marc N. Potenza

**Affiliations:** ^1^Yale School of Medicine, Yale University, New Haven, CT, United States; ^2^Department of Psychiatry, Yale School of Medicine, New Haven, CT, United States; ^3^Connecticut Mental Health Center, New Haven, CT, United States; ^4^Connecticut Council on Problem Gambling, Wethersfield, CT, United States; ^5^Child Study Center, Yale School of Medicine, New Haven, CT, United States; ^6^Department of Neuroscience, Yale University, New Haven, CT, United States; ^7^Wu Tsai Institute, Yale University, New Haven, CT, United States

**Keywords:** ADHD, qualitative research, lived experiences, adult, emotional dysregulation, attentional dysregulation

## Abstract

Attention-deficit/hyperactivity disorder (ADHD) is a common condition that frequently persists into adulthood, although research and diagnostic criteria are focused on how the condition presents in children. We aimed to review qualitative research on lived experiences of adults with ADHD to characterize potential ADHD symptomatology in adulthood and provide perspectives on how needs might be better met. We searched three databases for qualitative studies on ADHD. Studies (*n* = 35) in English that included data on the lived experiences of adults with ADHD were included. These studies covered experiences of receiving a diagnosis as an adult, symptomatology of adult ADHD, skills used to adapt to these symptoms, relationships between ADHD and substance use, patients’ self-perceptions, and participants’ experiences interacting with society. Many of the ADHD symptoms reported in these studies had overlap with other psychiatric conditions and may contribute to misdiagnosis and delays in diagnosis. Understanding symptomatology of ADHD in adults may inform future diagnostic criteria and guide interventions to improve quality of life.

## Introduction

Attention-deficit/hyperactivity disorder (ADHD) has an estimated prevalence of 7% among adults globally (1). ADHD has historically been considered a disorder of childhood; however, 40–50% of children with ADHD may meet criteria into adulthood (2). Diagnostic criteria for ADHD include symptoms of inattention, hyperactivity, and impulsiveness present since childhood (3). These criteria are largely based on presentations in children, although diagnostic criteria have changed over time to better but not completely encompass considerations of experiences of adults (3, 4).

Although adult ADHD is highly treatable with stimulant medication (5), adults with ADHD often have unmet needs. Substance use disorders (SUDs) are approximately 2.5-fold more prevalent among adults with versus without ADHD (6, 7). Adults with ADHD are particularly likely to be incarcerated, with 26% of people in prison having ADHD (8). As diagnosis of ADHD has increased considerably in recent decades (9), there are likely many adults with ADHD who were not originally diagnosed as children. In more recent years, ADHD is still frequently underdiagnosed or misdiagnosed as other psychiatric conditions such as mood or personality disorders (10). Even when patients are diagnosed with ADHD as children, many patients lose access to resources when transitioning from child to adult health services (11) which may contribute to less than half of people with ADHD adhering to stimulant medication (12).

Non-pharmacological interventions such as cognitive behavioral therapy (CBT) have shown promise with helping adults manage their ADHD symptoms, although such symptoms are not completely ameliorated by therapy (13–15). A more thorough understanding of the symptoms adults with ADHD experience and the effects that these symptoms have on their lives may allow for more efficacious or targeted therapeutic interventions.

Qualitative research may provide insight into lived experiences, and findings from such studies may direct future research into potential symptoms and therapeutic interventions. The aim of this review is to describe the current qualitative literature on the lived experiences of adults with ADHD. This review may provide insight into the symptomatology of adult ADHD, identify areas where patient needs could be better met, and define gaps in understanding.

## Methods

### Search strategy

Using rapid review methodology (16), PubMed, PsychInfo, and Embase were searched on October 11th, 2021 with no date restrictions. The search terms included “ADHD” and related terms as well as “qualitative methods” present in the titles or abstracts. The full search (Supplementary Appendix 1) was conducted with the help of a clinical librarian. The search yielded 417 articles which were uploaded to Endnote X9 where 111 duplicates were removed. The remaining 307 articles were uploaded to Covidence Systematic Review Management Software for screening, with one additional duplicate removed. The search also yielded a previous review on the lived experiences of adults with ADHD (17). The ten articles present in this review were also uploaded to Covidence where two duplicates were removed resulting in 314 unique articles.

### Study selection

Studies reporting original peer-reviewed qualitative data on the lived experience of adults with ADHD, including mixed-methods studies, were eligible for inclusion. “Adult” was defined as being 18 years of age or older; studies that included adolescent and young adult participants were only included if results were reported separately by age. Studies that included some participants without ADHD were included if results were reported separately by diagnosis. Any studies with adult participants who were exclusively reflecting on their childhood experiences with ADHD were considered outside this study’s scope, as were studies on family members, medical providers, or other groups commenting on adults with ADHD. Articles could be from any country, but needed to have been published in English. Individual case studies were not included due to concerns with generalizability.

Twenty percent of titles and abstracts were screened by two reviewers for meeting the inclusion criteria. Studies were not initially excluded based on participants’ ages as many titles and abstracts did not specify age. One reviewer screened the remaining abstracts; a second reviewer screened all excluded abstracts. For full-text screening, ten articles were screened by both reviewers to ensure consistency. One reviewer screened the remaining articles; a second reviewer screened all excluded articles.

### Quality appraisal

Quality appraisal was completed by one reviewer using the Joanna Briggs Institute critical appraisal checklist for qualitative research (18). Half of included studies did not state philosophical perspectives, two-thirds did not locate researchers culturally or theoretically, nearly one-third did not include specific information about ethics approval, and only two studies commented on reflexivity (Supplementary Appendix 2). Given the varied quality appraisal results and the small body of literature, all studies were included regardless of methodological rigor.

### Data extraction

Data extracted included general study characteristics and methodology, participant characteristics (sample size, demographics, and country of residence), study aims, and text excerpts of qualitative results. Study characteristics were entered into a Google Sheets document. PDFs of all studies were uploaded into NVivo 12, and results sections were coded using grounded theory (19). One reviewer extracted and coded data; a second reviewed extracted data for thematic consistency.

## Results

### Study characteristics

**FIGURE 1:**
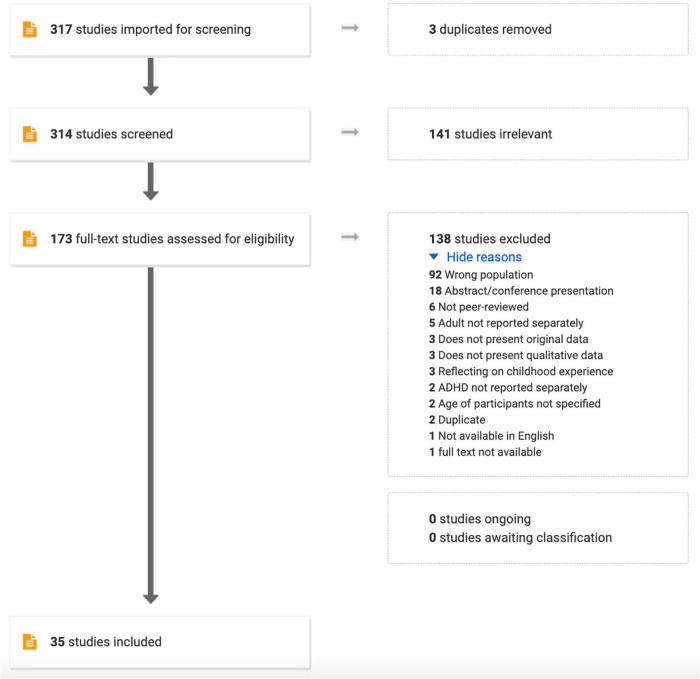
PRISMA flow diagram showing the search strategy for identifying qualitative studies on the lived experience of adult attention-deficit/hyperactivity disorder (ADHD).

One-hundred-and-seventy-three articles were deemed relevant in title and abstract screening. Of these, 35 were included after the full-text review (Figure 1). Articles were published between 2005 and 2021, and methodology mostly consisted of individual interviews (91%), with other studies utilizing focus groups (14%). Eight studies focused on young adults (18–35 years), and three were specific to older adults (>50 years). Two had exclusively male participants, and three had exclusively female participants. Nineteen were conducted in Europe, nine in North America, and three in Asia. No studies included participants from Africa, South America, or Oceania. In six studies, participants had current or prior SUDs, six studies focused on college students, four included participants diagnosed in adulthood, and two included highly educated/successful participants (Table 1).

**TABLE 1:** Article characteristics of included studies.

Author	Aim	Country	Method	*N*	Age, years^1^	Sex/gender	Race/ethnicity
Ando et al. (34)	How COVID-19 affects living conditions for adults receiving an adaptive coaching intervention	Japan	Interviews	4	20s–40s	50% male	–
Aoki et al. (25)	Experience of being diagnosed in adulthood	Japan	Interviews	12	20–60	50% male	–
Brod et al. (20)	Burden of illness of ADHD	Canada, France, Germany, Italy, Netherlands, United Kingdom, and United States	Focus groups and interviews	108		52% male	74% white, 3% black, 3% Hispanic, 13% mixed
Brod et al. (33)	Examine quality of life issues	United States	Focus groups and interviews	29	18–59	65% male	–
Canela et al. (46)	Skills and coping strategies used before diagnosis or treatment	Switzerland	Interviews	32	34% > 45	56% male	–
Canela et al. (48)	Opinions and attitudes toward testing and stimulant treatment of children with ADHD	Switzerland	Interviews	32	25% > 51; 22% 21–30	56% male	–
Ek et al. (42)	How adults with ADHD perform everyday activities	Sweden	Interviews	12	21–38	50% male	–
Goffer et al. (35)	Occupational experiences of college students	Israel	Interviews	20	25.4 (3.67)	35% male	–
Hansson Halleröd et al. (26)	Experience of being diagnosed in adulthood	Sweden	Interviews	21	32 (9)	48% male	–
Henry and Jones (31)	Experiences of women in late adulthood	United States	Interviews	9	>60	100% female	78% white 22% Hispanic
Kronenberg et al. (44)	Consequences of SUDs^2^ for everyday life	Netherlands	Interviews	11	43	73% male	–
Kronenberg et al. (30)	Process of recovery from SUDs^2^	Netherlands	Interviews	9	36 (29–54)	89% male	–
Kwon et al. (38)	Difficulties in university life	South Korea	Interviews	12	22.2 (20–29)	41% male	–
Lasky et al. (39)	Role of context in declining symptoms in adulthood for people diagnosed as children	7 North American sites	Interviews	125	24 (1.7)	76% male	72% white, 10% black, 12% mixed
Lefler et al. (27)	What is it like to be a college student and what resources are utilized	United States	Focus groups	36	18–39; median 20	66% male	88% white
Liebrenz et al. (53)	Perceptions of cigarette use	Switzerland	Interviews	20	25–54	50% male	–
Liebrenz et al. (50)	Perceptions of smoking cessation and withdrawal	Switzerland	Interviews	12	25–53, 40	41% male	
Maassen et al. (21)	What do participants consider to be good healthcare	Netherlands	Focus groups	30	–	–	–
Matheson et al. (22)	Experience of diagnosis, treatment, and impairments between those diagnosed as children and adults	United Kingdom	Interviews	30	18–56+	43% male	80% white
Meaux et al. (32)	Factors that help and hinder college students	United States	Interviews	15	18–21	60% male	87% white
Meaux et al. (47)	Experience of stimulant use in college students diagnosed as children	United States	Interviews	15	18–21	60% male	86% white
Michielsen et al. (36)	How ADHD affects lives of older adults unaware of diagnosis	Netherlands	Interviews	17	67–86	41% male	–
Mitchell et al. (23)	Factors that delay diagnosis in children and why symptoms may emerge in adulthood	United States	Interviews	14	22–25	85% male	71% white
Mitchell et al. (51)	Relationship between substance use and disrupted emotional functioning in those diagnosed as children	United States	Interviews, mixed methods	70	21–26	74% male	77% white, 10% black, 10% mixed
Nehlin et al. (52)	Perception of substances in people with SUDs^2^	Sweden	Interviews	14	29.6 (7.8), median 25.5	42% male	–
Nordby et al. (49)	Experience of participating in a group-based intervention for goal management training	Norway	Interviews	10	21–49	70% male	–
Nystrom et al. (43)	Day to day life of people older than 50	Sweden	Interviews	10	51–74	70% female	–
Schreuer et al. (40)	Experiences of women in the workplace; strategies and accommodations used	Israel	Interviews	11	33.5 (6.61)	100% female	–
Schrevel et al. (41)	Perspectives, problems, and needs in daily life	Netherlands	Focus groups	52	43 (9.5)	46% male	–
Sedgwick et al. (45)	Positive aspects of ADHD among highly successful adults	United Kingdom	Interviews	6	30–65	100% male	
Toner et al. (24)	How people manage their symptoms	Australia	Interviews	10	30–57	100% male	–
Waite and Tran (28)	Experience of ethnic minority women in college	United States	Interviews	16	18–45	100% female	31% black 19% Hispanic 6% American Indian 13% Asian 25% other
Watters et al. (37)	Lived experience	Ireland	Interviews	11	20–54, mean 37	81% male	–
Weisner et al. (54)	Beliefs on ADHD, stimulant use, and substance use among those diagnosed as children	United States	Interviews	125	24.4 (1.18)	76% male	72% white 10% black 12% mixed
Young et al. (29)	Experience of diagnosis in adulthood	United Kingdom	Interviews	8	21–50 (mean 39)	50% male	–

^1^Ages not reported consistently across studies.

^2^Substance use disorder.

**FIGURE 2:**
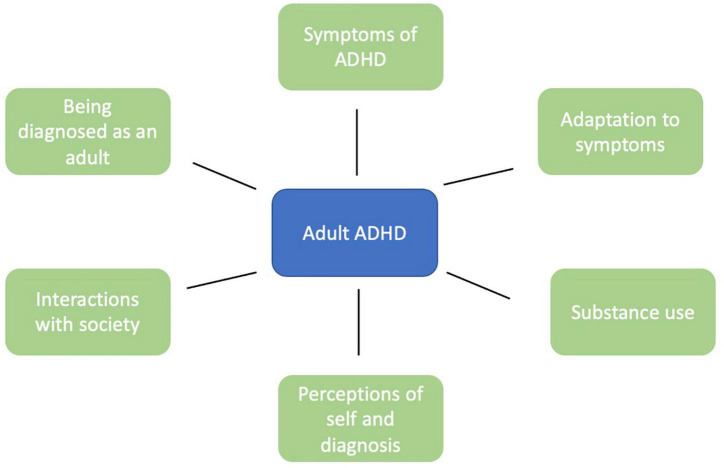
Schematic diagram of the domains of features linked to the lived experiences of adults with ADHD.

An overview of the identified themes is described in Figure 2, and Table 2 provides a summary of main findings. Several of the themes overlap with each other, and such areas are identified in the main text.

**TABLE 2:** Summary of results.

Adult diagnosis		Process of being diagnosed was laborious and initial misdiagnosis was frequent.
		Diagnosis commonly caused feelings of relief as well as identity changes including self-acceptance and emotional turmoil.
		Participants wished they had been diagnosed sooner in life.
Symptomatology of ADHD	Inattention	Attention was influenced by the environment and interest in the present task; participants did not experience a pervasive deficit of attention.
	Impulsivity	Resulted in risk-taking and impulsive speech.
	Hyperactivity	Less commonly reported; usually inner feelings of restlessness as opposed to physical hyperactivity.
	Chaos	Internal feelings of chaos as well as disorganized lives were common.
	Structure	Decreased structure in adulthood was difficult to manage.
	Emotions	Participants experienced emotional dysregulation, unpleasant emotions, and difficulty recognizing emotions.
	Positive aspects of ADHD	ADHD was seen as promoting spontaneity, creativity, energy, and resilience.
Adapting to symptoms	Coping skills	Organization strategies, environmental modifications, physical activity, and awareness of diagnosis were seen as helpful.
	Medications	Stimulants helped with achieving goals and increasing productivity.
		Adverse effects included difficulties socializing, somatic effects, changes in emotion, and rebound symptoms.
	Outside support	Workplace and school accommodations were helpful.
		Individual therapy such as CBT was seen as helpful, although it needed to be more tailored to ADHD.
		Support groups were desired to help build community and learn coping skills.
Substance use	Reasons for substance use	Self-medication and impulsive decision-making contributed to substance use.
	Quitting	Although a difficult process, quality of life improved after discontinuing substances.
	Stimulants and other substances	Stimulants were seen as both a protective factor against substance use and as increasing risk of substance use by different participants.
Perceptions of self and diagnosis	Self-esteem	Low self-esteem due to external pressures was common, although self-esteem often improved in adulthood.
	Views of ADHD	Some viewed ADHD as a difference instead of a disability; others found the diagnosis limiting.
		There were mixed opinions regarding whether participants wished ADHD could be cured.
Interactions with society	Relationships with others	Participants struggled with interpersonal relationships and feeling different from others.
	Outside perceptions of ADHD	Stigma about the legitimacy of adult ADHD was common; many did not disclose their diagnosis to others.
	Societal expectations	Failure to keep up with activities of daily living resulted in low self-esteem.
	Education and occupation	Underachievement was widespread; medications, accommodations, and tailoring tasks to personal interests were seen as helpful.
	Accessing services	Receiving medications, counseling, and appointments were difficult to navigate and often required self-advocacy.

### Adult diagnosis

Assessment and diagnosis of adult ADHD were reported as laborious and included prior misdiagnoses (20–22), lack of psychiatric resources (23), and physicians’ stigma regarding adult ADHD (24). Participants were often diagnosed only after their children were diagnosed (23, 24). However, after receiving a diagnosis, relief was commonly reported initially. Adults noted that receiving a diagnosis helped explain previously seemingly inexplicable symptoms and feelings of being different, and allowed for participants to blame themselves less for perceived shortcomings (24–31).

Identity changes were another reported finding after diagnosis, both positive and negative. Some participants reported experiencing existential questioning of their identities (25, 26); others reported feeling increased levels of self-awareness (26, 28). Some participants reported having initial doubts about the validity of their diagnoses (26, 28). Some reported experiencing emotional turmoil and concerns about the future (25, 26, 29). A commonly reported late step involved acceptance, both of themselves and their diagnoses, sometimes coupled with increased interest in researching ADHD (24, 25, 28, 29, 32). A ubiquitous finding was participant regret that they had not been diagnosed earlier, largely because of the many years they had gone without understanding their condition or receiving treatment (22, 24, 26–30). In one study, participants who had been diagnosed as children had better emotional control and self-esteem (33). No studies reported participant regret about their ADHD diagnosis.

### Symptomatology of attention-deficit/hyperactivity disorder

#### Inattention, impulsivity, and hyperactivity

Consistent with current diagnostic conceptualizations, difficulties with attention and concentration were described. These difficulties hindered completion of daily life tasks at home, school, and work (24, 27, 28, 32, 34–37). Some participants reported not experiencing a pervasive deficit of attention, but rather only struggling when the topic was not of personal interest and could sustain attention on interesting tasks for long periods of time (33, 38–42). Attention could be influenced by the environment; for example, attention worsened in distracting environments or improved in intense, stimulating environments (40, 41).

Impulsivity was widely reported and reflected in risk-taking including reckless driving, unprotected sex, and extreme sports (20, 24, 28, 33, 36, 43). Impulsive spending was noted (20, 36–38, 44). Impulsive speech (“blurting out”) was common and often led to strained interpersonal relationships (24, 32, 33, 36, 37, 40).

Fewer studies described participants’ struggles with hyperactivity, such as with staying still or not being constantly busy (24, 34, 36). Hyperactivity was reported as an internal symptom by some participants, noted as inner feelings of restlessness (22, 36, 37, 39), or described as resulting in excessive talking (36). This more subtle hyperactivity was mostly reported by women or older adults.

#### Chaos, lack of structure, and emotions

Living in chaos was often reported, whether involving internal feelings of being unsettled (28), or external aspects such as turbulent schedules or disorganized living spaces (22, 24, 27, 36). Participants often struggled with maintaining structure in daily routines, resulting in irregular sleeping and eating, difficulty completing household tasks, and strained social lives (36–38, 43, 44). Increased autonomy in adulthood was often perceived as difficult to manage compared to more highly structured childhoods.

Although lacking from current diagnostic criteria, emotional dysregulation was often noted. Participants reported experiencing extreme emotional reactions to interpersonal conflicts such as terminations of romantic relationships or receiving negative feedback at work (24, 34, 38, 40). Negative feelings of anxiety and agitation were common (22, 24, 29, 31, 33, 34, 36, 38, 44), as was difficulty with controlling, recognizing, naming, and managing emotions (30, 40, 41, 44). One study noted that emotional lability has positive aspects since participants’ emotional highs were higher (45).

#### Positive aspects of attention-deficit/hyperactivity disorder

Not all aspects of ADHD were perceived as negative. Impulsivity was reported by some as fun and spontaneous (26, 37, 45), struggles with attention were reported as promoting creativity and motivating focus on details (21, 33, 40, 41, 45), and hyperactivity was described as providing energy to pursue one’s passions (40, 45). Learning to live with ADHD-related impairments was reported as promoting resilience and humanity (45), and increased tendencies to keep calm in chaotic settings (40). Ability to maintain focus for extended periods on topics of personal interest was sometimes seen as helpful, although unpredictable (33).

### Adapting to symptoms

#### Coping skills

Participants reported compensatory organizational strategies that increased structure in their daily lives. Creating regimented sleeping, eating, working, and relaxing schedules (30, 35, 42, 44, 46), and keeping to-do lists or using reminder apps (24, 32, 37, 40, 42, 46) were frequently-reported strategies. Some participants reported thriving without formal structure while working from home since they were able to maintain daily routines and were free from distractions (34).

Participants reported being able to adjust their environment to best suit their needs, whether that be decreasing distracting stimulation (32, 46) or cultivating a highly stressful and stimulating environment (39). Creating space for physical activity was reported as a helpful outlet for hyperactivity (24, 33, 39, 43, 46). Having awareness of their diagnosis allowed newly-diagnosed participants to attribute their symptoms to their disorder, thereby decreasing self-blame (24, 26, 32). In one study, participants engage in self-talk to modify their behavior (32). Participants reported implementing social skills to prevent interrupting others and adjusting their social circles to accommodate their symptoms (24, 35, 46).

Substance use was also described as a coping strategy, although there were also drawbacks associated with using substances. Such findings are discussed under “substance use.”

#### Medication

Stimulant medications were commonly used to help manage ADHD symptoms; participants reported that stimulants facilitated task prioritization, goal achievement, and productivity often to “life-changing” extents (22, 24–27, 29, 32, 35, 40, 46–48). Stimulants were sometimes reported as assisting with social and emotional functioning by promoting calmness (22, 24, 30, 40). Some participants took their medications on an as-needed basis, choosing to take them only when they had much work (20, 27, 32, 33, 47). In one study, participants reported feeling pressured to sell their medication, and in another, participants reported increasing their dosages to stay up all night in order to better complete school work (27, 47).

Participant ambivalence or hesitation to take stimulants was reported due to therapeutic and adverse effects. Reported adverse effects included “not feeling like oneself,” resulting in difficulties with socializing and creativity (22, 27, 35, 40, 47), somatic effects such as appetite suppression and insomnia (22, 27, 35, 40, 47), unpleasant emotions including irritability and numbness (35, 40, 47), and rebound symptoms and withdrawal side effects when the medications wore off (29, 47).

#### Outside support

Studies noted participants adapting to living with their symptoms by receiving formal accommodations at work and school. Reported workplace accommodations included reduction of auditory distractions and bosses who would provide organizational advice or extra reminders about due dates (24, 25, 40). Reported accommodations in college consisted of separate testing environments and extra time on examinations. However, inaccessibility of disability offices, limited willingness of professors to comply with accommodations, and lack of participant engagement with accommodations due to not wanting to seem different resulted in many participants not utilizing such resources (27, 32).

Individual therapy was reported as helpful for managing symptoms and acquiring self-knowledge, especially therapeutic interventions designed for ADHD and CBT (22, 23, 27, 41). However, some participants reported minimal benefits from seeing therapists who did not specialize in ADHD, and CBT was reported to need improvement to be specially tailored to adults with ADHD such as being more engaging or being reframed as ADHD coaching (22, 27, 33). Community care workers added structure to some participants’ lives and aided with motivation in one study (42).

In some studies, participants expressed desires to be involved with support groups for adults with ADHD in order to learn new coping skills and find community, but not knowing where to access such services (28, 40). Those who had participated in ADHD support or focus groups reported feeling validated and less isolated, as well leaving with improved strategies for symptom management (24, 31, 41, 49). Support was also reported in personal relationships. Having a supportive partner often helped participants tremendously with organization and life tasks, especially for men married to women (24, 43). A close friend or family member encouraging accountability and creating a sense of togetherness was viewed as advantageous (32, 42).

### Substance use and addiction

#### Reasons for substance use

The SUDs were commonly reported among adults with ADHD and often seen as a form of self-medication. In every study that discussed self-medication, participants reported using substances to feel calm and relaxed; substances included nicotine/tobacco, alcohol, marijuana, cocaine, and methamphetamine (20, 24, 32, 46, 50–52). Nicotine/tobacco, marijuana, ecstasy (MDMA), and methamphetamine were used to help improve focus, particularly before diagnosis and subsequent to stimulant treatment (20, 24, 32, 51, 52). Participants also reported using substances to help feel “normal” as they facilitated social interactions and helped complete activities of daily life (20, 50, 52). One study described college males’ experiences with video game addictions which resulted in neglecting schoolwork (32).

The tendencies of people with ADHD to make impulsive decisions were suggested as linking ADHD and substance use (20, 52). Substance use worsened ADHD symptoms, most notably impulsivity (44, 52). One study attributed high rates of substance use to participants with ADHD being less fearful and more rebellious than individuals without ADHD (50).

#### Quitting

Although discontinuing substance use was regarded as a difficult process with frequent relapses, participants considered their quality of life to improve after quitting (30, 44, 53). Nicotine withdrawal was reported to worsen ADHD symptoms, and participants desired smoking-cessation programs specifically tailored for those with ADHD (53). Even after discontinuation of substance use, participants reported difficulties accessing stimulant medication due to their substance-use histories (52).

#### Stimulants and use of other substances

Findings relating stimulant use and use of other substances were mixed. Prescription stimulant usage was reported as a protective factor against use of other substances. Participants who had previously been self-medicating reported that when they had been on stimulants, they did not need other substances to help them feel calm and focused (46, 47, 50, 52). Stimulants were reported to decrease cigarette cravings (50). In one study, a participant commented that her stimulant prescription generated a hatred of taking pills, which she reported subsequently prevented her from using drugs (54).

Some participants reported stimulant prescriptions as increasing risk of substance use. Some reported that stimulants directly increased nicotine cravings (50). Indirect connections were reported, such as feelings of social exclusion due to being labeled as medicated or due to participants feeling used to taking drugs since childhood (54). Other participants reported no connection between stimulant medication and use of other substances (50, 54).

### Perceptions of self and diagnosis

#### Self-esteem

Participants often reported experiencing low self-esteem which they attributed to feeling unable to keep up with work or school, being told they were not good enough by others, and frequently failing at life goals (24, 27–29, 33, 36, 37, 41, 43). Low self-image was typically worse in childhood and improved over time, especially after receiving a diagnosis (28, 36, 43). In one study, some participants did not see themselves as having any flaws despite repeatedly being told otherwise, possibly due to being distracted from the emotional impact of these remarks (29).

#### Views of attention-deficit/hyperactivity disorder

Some participants viewed ADHD as a personality trait or difference as opposed to a disorder or disability (31, 32, 39, 41, 45). Some participants reported finding the ADHD diagnosis limiting and not wanting the disorder to define who they were (27, 28). When asked if they would want their ADHD “cured” in one study, participants’ responses ranged from “definitively yes” to “definitely no.” Many reported feeling ambivalent as they described both positive and negative aspects of ADHD (20).

### Interactions with society

#### Relationships with others

Difficulties building and maintaining relationships with others were regularly reported. Participants reported that impulsivity hindered their social interactions due to their tendencies to make inappropriate remarks, engage in reckless behaviors, and agree to engagements without thinking through consequences, resulting in being associated with people to whom they did not want to be linked (20, 22, 32, 33, 36, 43). Reported organizational struggles contributed to participants frequently being late and having cluttered living spaces (24, 38). Participants reported misunderstanding social norms and hierarchies and being hesitant about starting conversations (28, 30, 40, 43). They reported feeling overwhelmed by others’ emotions and unsure how to respond to them (44). Some participants reported choosing to hide their ADHD diagnoses, and the resultant barrier made socializing feel exhausting (24). Participants reported that these factors made sustaining long-term relationships especially difficult (22, 31, 38, 43).

Feeling different from others was widely reported, most notably in childhood (20, 24, 27, 29, 31, 32). This experience was described as feeling misunderstood, like a misfit, abnormal, and/or like there was something wrong with them (20, 24, 27, 29, 33, 43, 45, 50). Participants reported consciously pretending to be normal as an attempt to fit in (28, 41). Some participants reported seeing themselves as more brave or rebellious than their peers, which sometimes resulted in positive self-images (24, 36, 50). A strong desire to advocate for “the underdog” in interpersonal relationships was described by some women (31). In one study, most participants did not describe feeling different from others, but reported having felt misunderstood as children (36).

Participants with ADHD who also had children diagnosed with ADHD reported that their approaches to their children’s diagnoses were shaped by their own ADHD experiences. Parents reported uniform support of diagnostic testing, although the best time for testing was not agreed-upon (26, 48). Opinions on starting their children on stimulants varied, ranging from enthusiastic support to viewing medication as a last resort, even among participants who had responded positively to stimulants themselves (48). Most participants reported supporting shared decision-making with the child.

#### Outside perceptions of attention-deficit/hyperactivity disorder

Participants reported their social networks often expressed preconceived notions about the diagnosis, such as ADHD being “fake” or restricted to children (27–29, 37, 41). Stigma about ADHD was reported as having prevented many from disclosing their diagnosis both personally and professionally (24, 26, 28, 29, 32). Increased awareness and education about ADHD were desired by participants to help them function better in society (28, 41).

#### Societal expectations

Some studies discussed participants’ difficulties with meeting societal expectations. Participants reported struggling to keep up with daily tasks such as maintaining their living spaces, paying bills and remembering to eat (28, 33, 35, 41). These difficulties were reported to result in exasperation, low self-esteem, and exhaustion (29, 33).

#### Education and occupation

Academic underachievement was widely reported; most studies focused on postsecondary education. Some participants reported having to try harder than their peers for the same results (28, 35), while others reported that they fell behind due to not putting in much effort (24, 27). Reports of low motivation to complete assignments until the last minute, as it then became easier to focus, led to missed deadlines (32, 35, 38). Participants reported difficulties paying attention in class (24, 27, 32, 35), struggling with reading comprehension (27, 32), and needing extra tutoring (24, 28). Participants reported these difficulties prevented them from “reaching their potential” as they were unable to complete advanced courses or degrees necessary for their careers of choice (20, 22, 31, 37, 39). A third of participants in one study noted that they did not struggle academically (31). Reported coping mechanisms for mitigating academic impairment included medications (35, 47), active engagement with materials facilitated by small class sizes or study groups (23, 35), and studying from home with fewer distractions (34). Formal academic accommodations are discussed under the outside support subheading of adapting to symptoms.

Occupational struggles were commonly reported, with many studies detailing participant underemployment or unemployment and high job-turnover rates (22, 31, 33, 37, 41, 43). Difficulties with punctuality and keeping up with tasks and deadlines were reported to generate tensions in the workplace (20, 22, 24, 33, 35, 39), and participants reported frequently being bored and unable to stay focused on their responsibilities, with noisy workplaces promoting distractibility (20, 24, 33, 35, 39, 40). Some studies noted difficulties understanding and navigating social hierarchies in the workplace (20, 40). In one study, participants reported feeling unable to maintain work-life balance, overworking until they felt burnt out (36). Working in fields of intrinsic interest, multitasking, and self-employment were reported strategies used to achieve occupational success (24, 31, 40). Having an understanding employer who could assist with task delegation and understand their needs was described as promoting positive workplace dynamics (25, 33, 40). Clearly defied roles and working with others helped some participants remain engaged in work (42). College students often reported part-time jobs as rewarding, with responsibilities helping them manage their academic pursuits (35).

#### Accessing services

Adults described difficulties accessing healthcare for ADHD. Most reported having to fight to receive a diagnosis and medication due to perceptions of stigma from physicians about adult ADHD (22). After diagnosis, participants often felt they did not receive adequate counseling or follow-up, especially when seeing general practitioners (22, 26). Many participants reported not seeing physicians regularly for medication management due to bureaucratic difficulties (21); college students reported often having their former pediatricians refill prescriptions without regular appointments (47). Many participants in one study had little knowledge of ADHD services available to them despite regular appointments (32).

## Discussion

This review characterizes the current literature on the lived experiences of adults with ADHD. This includes experiences of having been diagnosed as an adult, symptomatology of adult ADHD, skills used to adapt to ADHD symptoms, relationships between ADHD and substance use, individual perceptions of self and of having received ADHD diagnoses, and social experiences interacting in society.

Similar themes were noted in a previous review on lived experiences of adults with ADHD consisting of ten studies, three of which were included here (17). Such themes included participants feeling different from others, perceiving themselves as creative, and implementing coping skills. There were also other similar findings from a review of eleven studies on the experiences of adolescents with ADHD (55). Overlapping themes included participants feeling that ADHD symptomatology has some benefits, experiencing difficulties with societal expectations, emotions and interpersonal conflicts, struggling with identity and stigma, and having varying experiences with stimulants. The overlaps in findings from these two reviews suggest there are shared experiences between adolescents and adults with ADHD. Unique from previous reviews on lived experiences of people with ADHD are the present qualitative findings of experiences of having received diagnoses in adulthood, reflections on ADHD and substance use, occupational struggles, attention dysregulation, and emotional symptoms of ADHD.

The relationship between ADHD effects and poor occupational performance has been previously described. People with ADHD often struggle with unemployment and underemployment and functional impairment at work (56–58). The findings of this review suggest that adults with ADHD may benefit from workplace accommodations and from decreased stigma around adult ADHD.

Findings suggest that people with ADHD often experience attention dysregulation as opposed to attention deficits, *per se*. This notion builds on previous clinical observations (59) and quantitative literature (60, 61) documenting that adults with ADHD may hyperfocus on tasks of interest. These findings suggest that inattention does not fully capture the attentional symptoms of the condition and suggest a possible need for updated diagnostic criteria.

Emotional dysregulation was described by many studies in this review, and there were no studies in which participants denied struggling with emotions. These findings provide support for a conceptual model of ADHD that presents emotional dysregulation as a core feature of ADHD, as opposed to models stating that emotional dysregulation is a subtype of ADHD or simply that the domains are correlated (62). Debates exist regarding whether or not specific clinical aspects of disorders constitute core or diagnostic features (63). The DSM-5 and ICD-11 have viewed differently the criteria for specific disorders, including with respect to engagement for emotional regulation or stress-reduction purposes [e.g., behavioral addictions like gambling and gaming disorders, and other behaviors relating to compulsive sexual engagement (3, 64, 65)]. Because emotional dysregulation is often overlooked as being associated with ADHD, patients experiencing such symptoms may be mistaken for having other conditions such as mood or personality disorders. Appreciating the emotional symptoms of ADHD may help psychiatrists, psychologists, and social workers more accurately diagnose ADHD in adults and decrease misdiagnosis.

The recurrent themes of difficulty naming and recognizing emotions found here suggest that ADHD may be associated with alexithymia. One study found that 22% of adults with ADHD were highly alexithymic but their mean scores on the rating scale for alexithymia were not significantly different from controls (66). Parenting style, attachment features, and ADHD symptoms have been found to predict emotional processing and alexithymia measures among adults with ADHD (67). More research is needed into the relationship between ADHD symptoms and alexithymia.

There was considerable heterogeneity in wishes regarding cures for ADHD (suggesting both perceived benefits and detriments) and stimulant use being association with SUDs. From a clinical perspective, both points will be important to understand better. With regard to the latter, ADHD and SUDs frequently co-occur; one meta-analysis found that 23% of people with SUDs met criteria for ADHD (68). Furthermore, youth with ADHD are seven-fold more likely than those without to experience/develop SUDs; however, early treatment with stimulants appeared to decrease this risk (69). Understanding better motivations for substance use in adults with ADHD as may be gleaned through considering lived experiences may help decrease ADHD/SUD co-occurrence and improve quality of life.

This review highlights gaps in the qualitative literature on adult ADHD. Nearly all included studies took place in Europe, North America or Asia; there is a dearth of qualitative research on ADHD in the Global South. Although most studies did not report race, those that did often had a majority of White participants. Racial/ethnic disparities in ADHD diagnosis may contribute to the relatively low diversity of study participants (9), and such disparities are further reason to expand research focused on non-White individuals with ADHD. Most studies focused on young or middle-aged adults and most participants were male; more research is needed on how ADHD may impact older adults and other gender identities. Although long considered to disproportionately affect male children at approximately 3:1 (70), ADHD in adults has been reported to have gender ratios of 1.5:1 (71). Among the adult psychiatric population, some studies have found no gender difference in prevalence or up to a 2.5:1 female predominance (72). This finding suggests that women often may not receive diagnoses until adulthood and there may be strong links with other psychopathologies in women. The lived experience of women with ADHD should be further examined; this insight may help to understand why women often go undiagnosed and experience other psychiatric concerns.

Future qualitative studies should explore how ADHD symptoms change over the lifespan as this was not addressed in any of the included studies. There were very few findings relating to how adults with ADHD conceptualize the condition and how their diagnosis interacts with their identities. Some studies reported on difficulties adults with ADHD have with accessing services; further exploration is needed into how the medical community can better meet the needs of this population. Findings from this review may be used to inform future ADHD screening tools. The Adult ADHD Self-Report Scale (ASRS) is a widely used screening tool that covers symptoms of inattention, impulsivity, and hyperactivity (73). This review suggests that symptoms may be more expansive than what is included in the ASRS and that questions on attentional dysregulation and hyperfocusing, emotional dysregulation, internal chaos, low self-esteem, and strained interpersonal relationships could be tested for validity for inclusion. The Conners’ Adult ADHD Rating Scales (CAARS) includes questions on emotional lability and low self-esteem in addition to symptoms covered by the ASRS (74), although the scale has been found to have high false-positive and false-negative rates (75). Further studies are needed to develop screening tools that capture the lived experience of adults with ADHD while maintaining appropriate sensitivity and specificity. This review may also inform tailoring CBT and other therapeutic interventions for ADHD. For example, CBT may help develop skills for volitional hyperfocusing on productive tasks instead of feeling pulled away from daily activities.

This study has limitations. Being a rapid review, it was not an exhaustive search of the available literature and may have missed some relevant studies that would have been identified by a systematic search. The search strategy consisted of ADHD and qualitative research methods; studies that did not include “qualitative” in their titles or abstracts may not have been identified. This may explain why the previous review on the lived experiences of adults with ADHD (17) included studies not identified by this search. Although a formal quality appraisal was completed, all studies were included regardless of the quality assessment as to not further narrow the review. For example, studies were not excluded based on how they verified ADHD diagnosis as many studies did not specify if or how this was completed. Although restricting studies based on quality metrics may have made the present findings more robust, the amount of data that would have been excluded would have been considerable and may have resulted in omitting important findings. These variable quality metrics not only limit the findings of the present review, but also speak to limitations in the methodological rigor of qualitative research on adult ADHD.

Attention-deficit/hyperactivity disorder is a relatively common diagnosis among adults. Exploration of the lived experiences of adults with ADHD may illuminate the breadth of symptomatology of the condition and should be considered in the diagnostic criteria for adults. Understanding symptomatology of adults with ADHD and identifying areas of unmet need may help guide intervention development to improve the quality of life of adults with ADHD.

## Author contributions

CG and MP contributed to the conception of the review. CG and GM-G performed the abstract and full text screening. CG performed the data synthesis and wrote the first draft of the manuscript. GM-G, GA, KB, and MP contributed to the revising and editing the manuscript. All authors read and approved the submitted version.
