# Supplementary material for: The lived experiences of adults with attention-deficit/hyperactivity disorder: A rapid review of qualitative evidence
Source: Front Psychiatry. 2022 Aug 11;13:949321. doi: 10.3389/fpsyt.2022.949321 (PMC9403235; doi:10.3389/fpsyt.2022.949321)
Supplement: Supplementary file 1 [file Data_Sheet_1.docx]

**Appendix 1**

**Full search strategy**

Pubmed:

(("attention deficit and disruptive behavior disorders"[MeSH Terms]) OR (ADHD[Title/Abstract] OR "attention deficit"[Title/Abstract])) AND (("qualitative research"[MeSH Terms]) OR qualitative [Ti]))

Results: 198 articles

PsychInfo:

1: "attention deficit and disruptive behavior disorders".hw. or (ADHD.ti. or ADHD.ab. or "attention deficit".ti. or "attention deficit".ab.) Results: 37,438 articles

2: exp Qualitative Methods/ Results: 17980 articles

3: 1 and 2

Results: 40 articles

Embase:

1: "attention deficit and disruptive behavior disorders".hw. or (ADHD.ti. or ADHD.ab. or "attention deficit".ti. or "attention deficit".ab.) Results: 37438 articles

2: exp qualitative research/

3: 1 and 2

Results: 179 articles

**Appendix 2**

**Quality Appraisal**

| Lead Author | Year | Q1 | Q2 | Q3 | Q4 | Q5 | Q6 | Q7 | Q8 | Q9 | Q10 |
| --- | --- | --- | --- | --- | --- | --- | --- | --- | --- | --- | --- |
| Ando | 2021 | Y | Y | Y | Y | Y | N | N | Y | Y | Y |
| Aoki | 2020 | N | Y | Y | Y | Y | Y | N | Y | Y | Y |
| Brod | 2012 | N | Y | Y | Y | Y | N | N | Y | Y | Y |
| Brod | 2005 | N | Y | Y | Y | Y | Y | Y | Y | Y | Y |
| Canela | 2017 | N | Y | Y | Y | Y | Y | N | Y | Y | Y |
| Canela | 2017 | N | Y | Y | Y | Y | Y | N | Y | Y | Y |
| Ek | 2013 | N | Y | Y | Y | Y | N | N | Y | Y | Y |
| Goffer | 2020 | Y | Y | Y | Y | Y | N | N | Y | Y | Y |
| Hansson Hallerod | 2015 | Y | Y | Y | Y | Y | Y | N | Y | Y | Y |
| Henry | 2011 | N | Y | Y | Y | Y | N | N | Y | Y | Y |
| Kronenberg | 2014 | N | Y | Y | Y | Y | N | N | Y | Y | Y |
| Kronenberg | 2015 | Y | Y | Y | Y | Y | N | N | Y | Y | Y |
| Kwon | 2018 | N | Y | Y | Y | Y | N | N | Y | Y | Y |
| Lasky | 2016 | Y | Y | Y | Y | Y | N | N | Y | N | Y |
| Lelfer | 2016 | Y | Y | Y | Y | Y | Y | Y | Y | Y | Y |
| Liebrenz | 2016 | Y | Y | Y | Y | Y | N | N | Y | Y | Y |
| Liebrenz | 2014 | Y | Y | Y | Y | Y | Y | N | Y | Y | Y |
| Maassen | 2016 | N | Y | Y | Y | Y | N | N | Y | Y | Y |
| Matheson | 2013 | N | Y | Y | Y | Y | Y | N | Y | Y | Y |
| Meaux | 2009 | N | Y | Y | Y | Y | N | N | Y | Y | Y |
| Meaux | 2006 | N | Y | Y | Y | Y | N | N | Y | N | Y |
| Michielsen | 2018 | N | Y | Y | Y | Y | N | N | Y | N | Y |
| Mitchell | 2021 | N | Y | Y | Y | Y | N | N | Y | N | Y |
| Mitchell | 2018 | N | Y | Y | Y | Y | N | N | Y | N | Y |
| Nehlin | 2015 | Y | Y | Y | Y | Y | Y | N | Y | Y | Y |
| Nrdby | 2021 | Y | Y | Y | Y | Y | Y | Y | Y | Y | Y |
| Nystrom | 2020 | Y | Y | Y | Y | Y | Y | N | Y | Y | Y |
| Schreuer | 2017 | Y | Y | Y | Y | Y | N | N | Y | Y | Y |
| Schrevel | 2016 | Y | Y | Y | Y | Y | N | N | Y | Y | Y |
| Sedgwick | 2019 | Y | Y | Y | Y | Y | N | N | Y | Y | Y |
| Toner | 2006 | Y | Y | Y | Y | Y | N | N | Y | N | Y |
| Waite | 2010 | N | Y | Y | Y | Y | N | N | Y | Y | Y |
| Watters | 2018 | N | Y | Y | Y | Y | N | N | Y | Y | Y |
| Weisner | 2018 | N | Y | Y | Y | Y | N | N | Y | Y | Y |
| Young | 2008 | Y | Y | Y | Y | Y | Y | N | Y | N | Y |

Q1: Is there congruity between the stated philosophical perspective and the research methodology?

Q2: Is there congruity between the research methodology and the research question or objectives?

Q3: Is there congruity between the research methodology and the methods used to collect data?

Q4: Is there congruity between the research methodology and the representation and analysis of data?

Q5: Is there congruity between the research methodology and the interpretation of results?

Q6: Is there a statement locating the researcher culturally or theoretically?

Q7: Is the influence of the researcher on the research, and vice- versa, addressed?

Q8: Are participants, and their voices, adequately represented?

Q9: Is the research ethical according to current criteria or, for recent studies, and is there evidence of ethical approval by an appropriate body?

Q10: Do the conclusions drawn in the research report flow from the analysis, or interpretation, of the data?
